# Adapting COVID-19 Contact Tracing Protocols to Accommodate Resource Constraints, Philadelphia, Pennsylvania, USA, 2021

**DOI:** 10.3201/eid3002.230988

**Published:** 2024-02

**Authors:** Seonghye Jeon, Lydia Watson-Lewis, Gabriel Rainisch, Chu-Chuan Chiu, François M. Castonguay, Leah S. Fischer, Patrick K. Moonan, John E. Oeltmann, Bishwa B. Adhikari, Hannah Lawman, Martin I. Meltzer

**Affiliations:** Centers for Disease Control and Prevention, Atlanta, Georgia, USA (S. Jeon, G. Rainisch, F.M. Castonguay, L.S. Fischer, P.K. Moonan, J.E. Oeltmann, B.B. Adhikari, M.I. Meltzer);; Philadelphia Department of Public Health, Philadelphia, Pennsylvania, USA (L. Watson-Lewis, C.-C. Chiu, H. Lawman);; University of Montreal School of Public Health, Montreal, Quebec, Canada (F.M. Castonguay)

**Keywords:** COVID-19, contact tracing, cases averted, resource efficiency, staff hours, coronavirus disease, SARS-CoV-2, severe acute respiratory syndrome coronavirus 2, viruses, respiratory infections, zoonoses, Philadelphia, Pennsylvania

## Abstract

Because of constrained personnel time, the Philadelphia Department of Public Health (Philadelphia, PA, USA) adjusted its COVID-19 contact tracing protocol in summer 2021 by prioritizing recent cases and limiting staff time per case. This action reduced required staff hours to prevent each case from 21–30 to 8–11 hours, while maintaining program effectiveness.

Case investigation and contact tracing (CICT) were among the primary nonpharmaceutical interventions for COVID-19 before vaccines became widely available. Previous studies estimated that CICT played an important role in mitigating the COVID-19 pandemic in the United States (*1*,*2*). However, CICT programs were resource-intensive and required trained personnel, testing capacity, and technology to support successful implementation (*3*,*4*). Health departments had to make decisions about how to best allocate limited resources to CICT and other competing mitigation strategies, such as vaccination, testing programs, and community outreach.

Because of a surge in cases associated with the SARS-CoV-2 Delta variant (B.1.617.2) during summer 2021 (*5*) and the redirection of staff hours from CICT to other activities, the Philadelphia Department of Public Health (PDPH; Philadelphia, PA, USA) adjusted its existing CICT protocol on August 18, 2021. The new protocol prioritized cases with the most recent specimen collection dates rather than on the basis of time registered in the surveillance system. In addition, instead of making multiple attempts to reach case-patients and contacts within ≈4 days, staff made 1 attempt to reach each case-patient and contact. The new protocol prioritized persons in the early stages of infection, aiming to prevent secondary transmission by allocating resources more effectively. In addition, by limiting the time allocated to each case, CICT staff could expand their reach to more persons. This redistribution of staff resources also supported the redirection of staff to other important response efforts.

## The Study

To assess the effect of the CICT protocol change, we defined two 8-week evaluation periods; period 1 was before the CICT protocol change (June 23–August 17, 2021), and period 2 was after the protocol change (September 1–October 26, 2021) (Figure). We employed a 2-week gap between the 2 periods to allow sufficient time for the effects of the new protocol to be reflected in reported cases. PDPH routinely collected the daily number of new COVID-19 cases (*6*), daily vaccination records (*6*), and CICT program metrics (*7*), including staff hours (Table 1). PDPH had a separate team responsible for overseeing contact tracing in select high-risk groups, such as nursing homes and other congregate living facilities; the effect of that team is not considered in the analysis. The PDPH Institutional Review Board determined that this work did not constitute human subjects research and was therefore not subject to institutional review board review.

**Figure:**
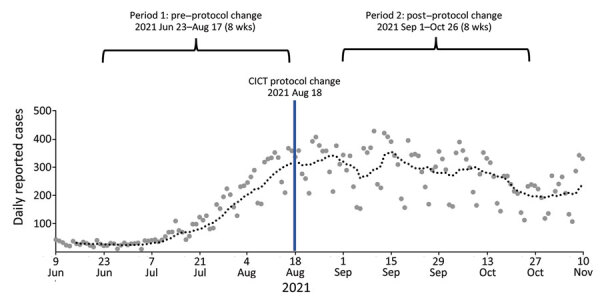
Daily reported COVID-19 cases and 2 evaluation periods before and after CICT protocol change, Philadelphia, Pennsylvania, June–November 2021. The large dots represent daily case counts, and the dotted line represents the 7-day moving average case count. CICT, case investigation and contact tracing.

**Table 1:** COVID-19 incidence, reported CICT program metrics, and CICT staff hours before and after CICT protocol change, Philadelphia, Pennsylvania, USA, 2021*

Characteristic	Period 1, before protocol change	Period 2, after protocol change
Evaluation dates	Jun 23–Aug 17	Sep 1–Oct 26
COVID-19 incidence		
Mean daily incidence, cases/100,000 persons†	9	18
Total no. reported cases	7,544	15,681
% Population fully vaccinated	58	65
CICT program performance metrics		
No. case-patients reached for interviews‡	5,685	9,351
No. case-patients who completed interviews (% all case-patients)	3,172 (42)	4,537 (29)
No. interviewed case-patients naming >1 contact	852	1,074
No. contacts identified	1,922	2,375
No. contacts notified	1,372	1,853
No. contacts monitored§	883	1,234
Timing of case-patient interview, days after specimen collection¶	3	2
Timing of contact notification, days after specimen collection#	4	3
CICT staff hours		
Average no. CICT staff per week	83	85
Total staff hours over the 8-wk period**	19,890	12,788

*CICT, case investigation and contact tracing. †Mean daily incidence for each of the 8-week evaluation periods. ‡Include case-patients who completed interviews, those who were reached but refused interview, and those who were reached but were unable to be interviewed because of other reasons (e.g., incarcerated, deceased, and language barriers). §Contacts who agreed to share symptom updates with the health department through text or phone calls. ¶Reported median days from specimen collection to positive test results reported to health departments. #Reported median days from specimen collection to contact notification. **On average, CICT staff spent 80% of their work (i.e., 30 h/wk) dedicated to CICT during period 1 and 50% during period 2 (i.e., 18.75 h/wk).

We combined data collected by PDPH with the Centers for Disease Control and Prevention COVIDTracer modeling tool (https://www.cdc.gov/ncezid/dpei/resources/covid-tracer-Advanced-Special-edition.xlsm) to estimate cases averted before and after the protocol change. COVIDTracer is a spreadsheet-based tool that uses a susceptible–exposed–infectious–recovered epidemiologic model to illustrate the spread of COVID-19 and the effects of community interventions such as CICT (*8*). We measured CICT effectiveness by calculating the proportion of case-patients and contacts isolated or quarantined in response to PDPH’s CICT efforts and the number of days needed for them to enter isolation or quarantine (Table 2). We then estimated the combined effects of other community interventions, such as masking, social distancing, and vaccination, by fitting the model-generated cumulative case curve to the observed one. Finally, to simulate a scenario without CICT, we removed CICT's effects in the model and calculated the difference between this hypothetical curve and the reported cases as the cases averted by CICT (Appendix). 

**Table 2:** Calculated CICT effectiveness values and model-estimated CICT effectiveness before and after CICT protocol change, Philadelphia, Pennsylvania, USA, 2021*

Characteristic	Period 1, before protocol change	Period 2, after protocol change
Calculated CICT effectiveness values		
% Case-patients and contacts isolated because of CICT (range)†	17 (11.7–21.9)	10 (6.7–12.5)
Days from infection to isolation‡	9	8
Model-estimated CICT effectiveness		
No. cases averted by CICT	657–968	1,156–1,609
No. hospitalizations averted by CICT	16–24	28–40
% Disease prevalence averted by CICT	8.4–12.0	6.8–9.2
Average staff hours per case averted§	21–30	8–11
Average staff hours per 1% disease prevalence averted¶	1,661–2,358	1,397–1,892

*CICT, case investigation and contact tracing. †Including contacts who later become case-patients. Calculated as follows using the observed performance metrics (Table 1), assumed compliance with isolation and quarantine guidance among cases and contacts (Appendix Table 1), and an assumed *k* = 1.2: [(% case-patients interviewed × compliance) + *k* × % contacts identified × (% contacts monitored × compliance + % contacts notified but not monitored × compliance)] / (1 + *k*), where *k* is approximated from the effective reproduction number (R_t_), because undetected infected contacts will infect R_t_ additional persons on average. During the evaluation period, the average R_t_ in Philadelphia was 1.29 during periods 1 and 0.99 during period 2. If the assumed compliance was 100%, the estimated effectiveness could be as high as 26% for period 1 and 15% for period 2. ‡The average length of time from infection to isolation and quarantine between case-patients and contacts who later became case-patients. We assumed a 5-day presymptomatic period. We further assumed that interviewed case-patients and notified contacts began isolation and quarantine the day after their interactions with the health department (Appendix). §Calculated by dividing the total staff hours by the estimated number of cases averted by CICT. Lower value represents a more cost-effective program, given that it requires fewer staff hours to prevent each case. ¶Calculated by dividing the total staff hours by the estimated proportion of disease prevalence averted by CICT. Lower value represents a more cost-effective program, given that it requires fewer staff hours to prevent each percentage of disease prevalence.

The percentage of cases interviewed declined from 42% to 29% after the protocol change, mainly because of a doubling of reported cases in period 2 (Table 1). However, a larger absolute number of case-patients were interviewed in period 2, resulting in more contacts being notified and monitored. Notification speed improved; case-patient interviews and contact notifications occurred 1 day faster after the protocol change (Table 1). We estimated that the percentage of case-patients and contacts isolated or quarantined because of CICT decreased after the protocol change, from 17% (range 11.7%–21.9%) to 10% (range 6.7%–12.5%). These ranges reflect different levels of assumed compliance with isolation and quarantine recommendations (Appendix Table 1). However, the number of days after specimen collection needed to start case-patient isolation and contact quarantine improved by 1 day, decreasing from 9 to 8 days (Table 2).

CICT efforts averted an estimated 657–968 cases during June 23–August 17 (period 1) and 1,156–1,609 cases during September 1–October 26 (period 2) (Table 2; Appendix Table 2). The estimate ranges consider various time values for exposed persons to become infectious, accounting for circulating COVID-19 variants (Appendix). The higher number of cases averted in period 2 may be influenced by the higher prevalence (Table 1); a larger number of cases in the community increases the potential for averting additional cases. The estimates of averted cases represent ≈8.4%–12.0% of the total disease prevalence in period 1 and ≈6.8%–9.2% of the total disease prevalence in period 2 (Table 2; Appendix Table 2).

When we calculated the effect of the protocol change by estimating cases averted in period 2 by using the CICT effectiveness values from period 1, the new protocol resulted in 93–189 fewer cases averted than would have occurred if the protocol had not changed (Appendix Table 3). This result indicates that, during the evaluation period, the benefits of increased notification speed were not sufficient to fully offset the negative effects of the lower coverage. Of note, factors beyond the implementation of the CICT program, such as variations in staff experience and efficiency between the 2 periods, and inherent errors associated with case-patient interviews may have influenced the results.

Similar numbers of staff were assigned to the CICT program during the 2 periods (an average of 83 staff per week in period 1 and 85 staff per week in period 2). However, on average, staff spent 80% of their time on CICT during period 1 (totaling 19,890 hours) and 50% of their time on CICT in period 2 (totaling 12,788 hours), which allowed staff to assist with vaccinations, testing, and other emergency response activities (e.g., influx of refugees from Afghanistan). Although CICT averted relatively more disease cases before the protocol change, average staff hours per case averted decreased after the protocol change (21–30 vs. 8–11 hours per case averted) (Table 2).

## Conclusions

PDPH’s new CICT protocol exemplifies the tradeoffs public health agencies in resource-limited settings encounter while working to fulfill their missions. Under the new protocol, the proportion of disease cases averted because of CICT decreased. However, the new protocol reduced staff hours needed to prevent each additional case by 63%. Throughout both periods, the estimated number of disease cases averted by CICT was meaningful, reducing the potential caseload by an estimated 300–800/month, depending on case levels and protocol changes.

Prioritizing more recently tested case-patients and limiting staff hours dedicated to each case-patient and contact resulted in increased efficiency of the CICT program. The staff time saved by the protocol change (7,103 staff hours saved over an 8-week period) (Table 1) was directed toward other meaningful mitigation efforts as the response evolved, including vaccination, testing, and outreach services.

Although resource-intensive, the CICT program collected valuable surveillance data on contextual, demographic, occupational, and exposure trends related to COVID-19. Furthermore, the direct interactions between CICT staff and residents provided essential health information and resources, encouraging positive behavioral changes that prevented further community transmission (*9*,*10*). In addition, CICT has proven effective in controlling outbreaks of Middle East respiratory syndrome and Ebola (*11*) and will serve as an important tool for managing other infectious diseases with pandemic potential. The inherent value of CICT underscores the need to implement more resource-efficient strategies, such as those used in PDPH’s protocol change, to sustain the program during future pandemics.

Appendix Additional information on adapting COVID-19 contact tracing protocols to accommodate resource constraints, Philadelphia, Pennsylvania, USA, 2021.
