# Supplementary material for: Adapting COVID-19 Contact Tracing Protocols to Accommodate Resource Constraints, Philadelphia, Pennsylvania, USA, 2021
Source: Emerg Infect Dis. 2024 Feb;30(2):333–6. doi: 10.3201/eid3002.230988 (PMC10826771; doi:10.3201/eid3002.230988)
Supplement: Appendix — Additional information on adapting COVID-19 contact tracing protocols to accommodate resource constraints, Philadelphia, Pennsylvania, USA, 2021. [file 23-0988-Techapp-s1.pdf]

Article DOI: https://doi.org/10.3201/eid3002.230988

EID cannot ensure accessibility for supplementary materials supplied by authors. Readers who have difficulty accessing supplementary content should contact the authors for assistance.

Adapting COVID-19 Contact Tracing Protocols to Accommodate Resource Constraints, Philadelphia, Pennsylvania, USA, 2021

Appendix

Methods

We used the Centers for Disease Control and Prevention (CDC)'s COVIDTracer modeling tool to estimate cases and hospitalizations averted by case investigation and contact tracing (CICT) (*1*,*2*). COVIDTracer uses an epidemiologic model to illustrate the spread of COVID-19 and the impact of interventions such as vaccines, CICT, and other non-pharmaceutical interventions (NPIs) (*3*). One of the key inputs to the model, CICT program effectiveness, was derived using the collected performance metrics (Table 1 in main text, https://wwwnc.cdc.gov/EID/article/30/2/23-0988-T1.htm). These effectiveness inputs included the coverage (percentage of cases and contacts isolated and quarantined due to their interactions with the health department) and the timeliness (number of days required from infection to isolation/quarantine). When deriving these values, we assumed 70% of interviewed cases, 52% of monitored contacts, and 10% of notified but unmonitored contacts fully complied with isolation and quarantine guidance. These values were estimated using national averages (*4*) and data collected by Philadelphia's CICT program, including survey agreement rates and responses to compliance questions following isolation and quarantine periods. We assessed a range of CICT impact by varying levels of public compliance to isolation and quarantine guidance by ±20 percentage points from the baseline (Appendix Table 1).

We then simulated what might have occurred in the absence of CICT using the COVIDTracer modeling tool. We generated a hypothetical COVID-19 case curve excluding the

contributions of CICT while maintaining the effects of vaccines and other NPIs. The difference between the reported cases and the model-simulated curve was the estimated cases averted by CICT (Appendix Figure 1). We calculated the proportion of the disease burden averted by CICT by dividing the averted case estimate by the cumulative case total of this model-simulated curve. We calculated the number of hospitalizations averted by multiplying the averted cases by the age-stratified infection-to-hospitalization ratio (*5*,*6*), Appendix Table 8. Lastly, we compared the two periods by examining the average staff hours per each averted case and the staff hours required to increase the averted disease burden by one percentage point.

Readers can use the publicly available tool (https://www.cdc.gov/ncezid/dpei/resources/covid-tracer-Advanced-Special-edition.xlsm) and the instructions provided in Rainisch *et al*. (*2*) to replicate the analysis for their respective jurisdiction.

Calculating CICT Effectiveness

We defined the effectiveness of the CICT program in terms of coverage (% of cases and contacts isolated and quarantined due to the program) and timeliness (number of days from exposure to isolation/quarantine). These effectiveness values were calculated using field-based data, such as the proportion of cases that completed case interviews (Table 1), as well as assumed values, such as public compliance with isolation and quarantine guidelines (Appendix Table 1). We assumed that a certain proportion of confirmed cases are effectively isolated following case interviews. We further assumed that a certain proportion of contacts are quarantined either upon contact notification or through active monitoring.

We calculated the average proportion of cases and contacts isolated and quarantined by the CICT program as follows:

$$\frac{(\text{\% Cases interviewed} * x_1) + k * \left(\text{\% Contacts identified} * (\text{\% Contacts monitored} * x_2 + \text{\% Contacts notified} * x_3)\right)}{(1+k)}$$

Here, x_1 represents the % of interviewed cases that isolated, x_2 represents the % of monitored contacts that quarantined, and x_3 represents the % of notified (but not monitored) contacts that quarantined. The multiplier k accounts for the expectation that the known case count represents just a fraction of the total secondary cases during our study period since undetected infected contacts would have further infected additional individuals. Therefore, we

used an approximation of the effective reproduction number (R_e) during our study period for the value of k: $k = 1.2$. If k>1 (indicating an outbreak is growing), the proportion of contacts identified has a larger impact on the overall CICT effectiveness compared to the proportion of cases interviewed. Conversely, if k<1 (indicating an outbreak is waning), the proportion of cases interviewed has a larger impact on the overall CICT effectiveness. During the evaluation period, the average R_e in Philadelphia was 1.29 and 0.99 during Periods 1 and 2, respectively. Therefore, using a single value of $k = 1.2$ was deemed sufficient as a proxy over the short period of time we analyzed.

The number of days from exposure to isolation/quarantine was determined by calculating the average number of days to case isolation and contact quarantine. We assumed that cases experience a 5-day pre-symptomatic period (*7*,*8*). To obtain the number of days from symptom onset to case interview, we added the reported *"Average days from symptom onset to specimen collection"* and the *"Average days from specimen collection to case interview"*. Additionally, we assumed that confirmed cases begin isolation the day after their interview (i.e., we added 1 to the total obtained above).

For contacts, we assumed they begin quarantine the day after receiving exposure notification from their health department. Since information on the actual dates of exposure for contacts was not available, we assumed that these individuals' exposures occurred at the midpoint of their potential exposure window (in days). We identified the earliest date in this window as the first day of infectiousness among cases to which contacts were exposed. We identified the latest possible exposure as the date the cases exposing them were interviewed by the health department (because they began isolation the next day). To calculate the number of days from contacts' exposure to their quarantine, we took the average of the maximum days a contact was infected and the fewest days the contact could be infected and weighted each day span by the case's infectiousness on each of the possible exposure days. Appendix Figure 2 illustrates the timing of exposure to isolation/quarantine for Philadelphia before the CICT protocol change, based on the aforementioned assumptions and the reported CICT performance metrics.

Defining the Susceptible Population and Accounting for Vaccination and Waning Immunity

The COVIDTracer modeling tool requires inputs to define the susceptible population. Individuals can be protected against infection through either vaccination or prior infection; however, immunity wanes over time. We assumed that both naturally acquired and vaccine-induced immunity last for 180 days. We also assumed no partial immunity (i.e., individuals are either fully protected or fully susceptible) during the evaluation period. We further assumed the likelihood of getting vaccinated is the same among the previously infected and uninfected individuals.

Based on these assumptions, we estimated the "fully protected" population as follows:

- Those fully vaccinated within 180 days of the evaluation period's start date
- Individuals who received a booster dose
- Those who were vaccinated 180 days ago or more (and thus lost immunity), but infected within 180 days
- Individuals who were unvaccinated but were infected within 180 days

The susceptible population is calculated by subtracting the "fully protected" population from the city's total population.

Epidemiologic Parameters for Delta Surge

The Delta variant accounted for ≈80% of all cases in both evaluation periods (6/23/21 – 10/26/21) in Philadelphia (*9*). Since the basic reproductive number (R_0) for the Delta variant was greater than that of the original SARS-CoV-2 strain (*10*), we used a weighted average for R_0 to account for the infectiousness of all variants in circulation as follows:

R_0 = 80% * R_0 for Delta + 20% * R_0 for other variants = 80% * 5.0 + 20% * 2.5 = 4.5.

Those infected with the Delta variant also appear to have a shorter latent period (days from exposure to being infectious), becoming infectious as early as 2 days post-exposure, compared to 3 days among those infected with variants in circulation before Delta's dominance (*11*,*12*). Without commensurate improvements in the speed of contact notification, a shorter latent period will contribute to a diminished impact from CICT, as infected individuals can transmit the virus more quickly before the health department could reach and isolate them. Therefore, to account for both the circulation of the Delta variant and other variants, we

estimated the impact of CICT (cases and hospitalizations averted) under two scenarios: 1) cases become infectious 2 days post-exposure, and 2) cases become infectious 3 days post-exposure. The former scenario provided a lower-bound estimate of CICT impact, while the latter provided an upper-bound estimate.

Extended Results

Sensitivity Analysis: Isolating effects of the protocol change

The two evaluation periods differed in various factors that could impact the performance of the CICT program. One notable difference was the mean daily incidence of COVID-19, which was twice as high during Period 2 due to the surge associated with the increased circulation of the Delta variant. In Period 2, the daily incidence was 18 cases per 100,000 population, while in Period 1, it was 9 cases per 100,000 population (Table 1).

To evaluate the isolated effects of the protocol change, we estimated the number of cases and hospitalizations averted in Period 2 (post-protocol change) assuming that the CICT protocol and its effectiveness remained unchanged from Period 1. Our analysis shows that the new protocol resulted in 93–189 fewer cases averted than would have occurred if the protocol had not changed (Appendix Table 3). This indicates that, during the evaluation period, the benefits of increased notification speed were not sufficient to fully offset the negative effects of the lower coverage.

Sensitivity Analysis: Potential effects of increased or decreased compliance with isolation and quarantine guidelines

If public compliance with isolation and quarantine guidelines was different than what we assumed in our baseline scenario (Appendix Table 1), the estimated number of cases and hospitalizations averted by CICT could have been 29% lower (low compliance) or 30% greater (high compliance) than the baseline scenario (Appendix Tables 4, 5).

COVIDTracer Modeling Tool, Overview and Assumptions

COVIDTracer is a spreadsheet-based tool that utilizes a Susceptible-Exposed-Infectious-Recovered (SEIR) epidemiologic model to illustrate the spread of a pathogen, the resulting disease, and the effects of interventions in a user-defined population (*3*). Interested readers can download the tool and enter input values of their choosing, exploring scenarios and assumptions

beyond those covered in this manuscript. The tool can be accessed through the following link: https://www.cdc.gov/ncezid/dpei/resources/covid-tracer-Advanced-Special-edition.xlsm.

To simulate the clinical progression and transmission of disease using COVIDTracer, we used the following definitions and assumptions. A "case" was defined as an individual who had been exposed, infected, and subsequently became infectious, regardless of the presence of clinical symptoms. We assumed that cases do not infect others for the first 3 days after infection. From days 4 to 5 post-infection, cases are pre-symptomatic but capable of shedding virus to infect others (*7*,*8*,*13*). From days 6 to 14, the infected individuals may experience symptoms and continue to shed virus, although the risk of onward transmission is relatively low during days 11 to 14. The complete infectivity distribution is outlined in Appendix Table 6. We assumed that ≈40% of cases were asymptomatic from days 6 to 14 but still posed a risk of onward transmission equivalent to 75% of symptomatic cases (Appendix Table 7) in the absence of vaccines or other non-pharmaceutical interventions (NPIs) (*13*). The model assumed homogeneous mixing among individuals and did not account for any age- or location-based heterogeneities in transmission (such as within and between households or schools), or variations in the effectiveness of vaccines and other NPIs over the study period. Furthermore, the tool used a deterministic model that did not account for uncertainties around parameters. Users are encouraged to alter the default parameter values and conduct sensitivity analyses to assess the impact of these assumptions (for reference, see (*10*,*14*) for a range of R_0 values).

References

1. Jeon S, Rainisch G, Lash RR, Moonan PK, Oeltmann JE Jr, Greening B Jr, et al.; Contact Tracing Impact Group. Estimates of cases and hospitalizations averted by COVID-19 case investigation and contact tracing in 14 health jurisdictions in the United States. J Public Health Manag Pract. 2022;28:16–24. PubMed https://doi.org/10.1097/PHH.0000000000001420

2. Rainisch G, Jeon S, Pappas D, Spencer KD, Fischer LS, Adhikari BB, et al. Estimated COVID-19 cases and hospitalizations averted by case investigation and contact tracing in the US. JAMA Netw Open. 2022;5:e224042. PubMed https://doi.org/10.1001/jamanetworkopen.2022.4042

3. Adhikari BB, Arifkhanova A, Coronado F, Fischer LS Jr. BG, Jeon S, et al. COVIDTracer Advanced: a planning tool to illustrate the resources needed to conduct contact tracing and monitoring of coronavirus disease 2019 (COVID-19) cases and the potential impact of community interventions

and contact tracing efforts on the spread of COVID-19 2020 [cited 2023 Jul 1]. https://www.cdc.gov/coronavirus/2019-ncov/php/contact-tracing/COVIDTracerTools.html

4. Oeltmann JE, Vohra D, Matulewicz HH, DeLuca N, Smith JP, Couzens C, et al. Isolation and quarantine for COVID-19 in the United States, 2020–2022. Clin Infect Dis. 2023;77:212–9. PubMed https://doi.org/10.1093/cid/ciad163

5. Bialek S, Boundy E, Bowen V, Chow N, Cohn A, Dowling N, et al.; CDC COVID-19 Response Team. Severe outcomes among patients with coronavirus disease 2019 (COVID-19)—United States, February 12–March 16, 2020. MMWR Morb Mortal Wkly Rep. 2020;69:343–6. PubMed https://doi.org/10.15585/mmwr.mm6912e2

6. Wu SL, Mertens AN, Crider YS, Nguyen A, Pokpongkiat NN, Djajadi S, et al. Substantial underestimation of SARS-CoV-2 infection in the United States. Nat Commun. 2020;11:4507. PubMed https://doi.org/10.1038/s41467-020-18272-4

7. He X, Lau EHY, Wu P, Deng X, Wang J, Hao X, et al. Temporal dynamics in viral shedding and transmissibility of COVID-19. Nat Med. 2020;26:672–5. PubMed https://doi.org/10.1038/s41591-020-0869-5

8. Ferretti L, Wymant C, Kendall M, Zhao L, Nurtay A, Abeler-Dörner L, et al. Quantifying SARS-CoV-2 transmission suggests epidemic control with digital contact tracing. Science. 2020;368:eabb6936. PubMed https://doi.org/10.1126/science.abb6936

9. University of Pennsylvania Perelman School of Medicine. SARS-CoV-2 variants circulating in the Delaware River Valley tracked by surveillance sequencing. [cited 2023 Jul 1]. https://microb120.med.upenn.edu/data/SARS-CoV-2

10. Liu Y, Rocklöv J. The reproductive number of the Delta variant of SARS-CoV-2 is far higher compared to the ancestral SARS-CoV-2 virus. J Travel Med. 2021;28:taab124. **PMID 34369565**

11. Li B, Deng A, Li K, Hu Y, Li Z, Shi Y, et al. Viral infection and transmission in a large, well-traced outbreak caused by the SARS-CoV-2 Delta variant. Nat Commun. 2022;13:460. PubMed https://doi.org/10.1038/s41467-022-28089-y

12. Wang Y, Chen R, Hu F, Lan Y, Yang Z, Zhan C, et al. Transmission, viral kinetics and clinical characteristics of the emergent SARS-CoV-2 Delta VOC in Guangzhou, China. EClinicalMedicine. 2021;40:101129. PubMed https://doi.org/10.1016/j.eclinm.2021.101129

13. Centers for Disease Control and Prevention. COVID-19 pandemic planning scenarios. 2021 Mar 19 [cited 2023 Jul 1]. https://www.cdc.gov/coronavirus/2019-ncov/hcp/planning-scenarios.html

14. Ge Y, Martinez L, Sun S, Chen Z, Zhang F, Li F, et al. COVID-19 transmission dynamics among close contacts of index patients with COVID-19: a population-based cohort study in Zhejiang Province, China. JAMA Intern Med. 2021;181:1343–50. PubMed https://doi.org/10.1001/jamainternmed.2021.4686

15. Centers for Disease Control and Prevention. COVID data tracker [cited 2023 Jul 1]. https://covid.cdc.gov/covid-data-tracker

Appendix Table 1. Assumed levels of public compliance with isolation and quarantine guidelines

	Isolation/Quarantine Compliance		
Case and contact categories	Low	Most Likely	High
Confirmed Cases that completed case interviews	50%	70%	90%
Contacts that are notified and monitored	32%	52%	72%
Contacts that are notified but not monitored	5%	10%	30%

Appendix Table 2. Estimated COVID-19 cases and hospitalizations averted by case investigation and contact tracing in Philadelphia, by period analyzed and assumed number of days for cases to become infectious.

		Number Averted (Percent of Cumulative Cases)	
Days for cases to become infectious	Health outcome	Period 1[a] 6/23–8/17	Period 2[b] 9/1–10/26
2 d post-exposure[c]	Cases	657 (8.4%)	1,156 (6.8%)
	Hospitalizations[d]	16 (8.4%)	28 (6.8%)
3 d post-exposure	Cases	968 (12.0%)	1,609 (9.2%)
	Hospitalizations[d]	24 (12.0%)	40 (9.2%)

[a] Period 1: Before the CICT protocol change
[b] Period 2: After the CICT protocol change
[c] Two studies found that the Delta variant may have a shorter latent period (days from exposure to being infectious) and that individuals infected with delta may become infectious as early as 2 d post-exposure, compared to 3 d for non-Delta variants (*11,12*).
[d] Number of hospitalizations averted is calculated by multiplying the estimated number of averted cases by the infection-to-hospitalization rate (Appendix Table 8). Therefore, the percent reduction in hospitalizations is identical to the percent reduction in cases.

Appendix Table 3. Estimated averted cases and hospitalizations during Period 2 (September 1 – October 26, 2021) attributed to the change in CICT protocol[a] of limiting case and contact outreach to one attempt.

Days for cases to become infectious	Health outcome	Difference in Number[b] averted due to protocol change
2 d post-exposure	Cases	−189
	Hospitalizations[c]	−5
3 d post-exposure	Cases	−93
	Hospitalizations[c]	−2

[a] Protocol change fully implemented on August 18.
[b] Estimated by using the reported CICT metrics from Period 1 (before protocol change): 17% of cases and contacts were effectively isolated (versus 10%) and took 9 d to do so (as opposed to 8 d).
[c] Number of hospitalizations averted is calculated by multiplying the estimated number of averted cases by the infection-to-hospitalization rate (Appendix Table 8). Therefore, the percent reduction in hospitalizations is identical to the percent reduction in cases.

Appendix Table 4. Estimated cases and hospitalizations averted by case investigation and contact tracing with varying levels of public compliance with isolation and quarantine guidelines (as per Appendix Table 2) during Period 1 (June 23 – August 17, 2021), pre-protocol change, Philadelphia.

		Number Averted (%)		
Days from cases to become infectious		Low Compliance	Most Likely	High Compliance
2 d post-exposure	Cases	466 (6.0%)	657 (8.4%)	854 (11.0%)
	Hospitalizations	11 (6.0%)	16 (8.4%)	21 (11.0%)
3 d post-exposure	Cases	689 (8.5%)	968 (12.0%)	1,252 (15.5%)
	Hospitalizations	17 (8.5%)	24 (12.0%)	31 (15.5%)

Note. Estimated by assuming different levels of compliance among interviewed cases and notified/monitored contacts, as described in Appendix Table 1.

Appendix Table 5. Estimated cases and hospitalizations averted by case investigation and contact tracing with varying levels of public compliance with isolation and quarantine guidelines (as per Appendix Table 2) during Period 2 (September 1 – October 26, 2021), post-protocol change, Philadelphia.

		Number Averted (%)		
Days from cases to become infectious		Low Compliance	Most Likely	High Compliance
2 d post-exposure	Cases	819 (4.8%)	1,156 (6.8%)	1,503 (8.8%)
	Hospitalizations	20 (4.8%)	28 (6.8%)	37 (8.8%)
3 d post-exposure	Cases	1,144 (6.5%)	1,609 (9.2%)	2,085 (11.9%)
	Hospitalizations	28 (6.5%)	40 (9.2%)	51 (11.9%)

Note. Estimated by assuming different levels of compliance among interviewed cases and notified/monitored contacts, as described in Appendix Table 1.

Appendix Table 6. Daily percentage risk of transmission by infectiousness state and clinical symptoms.

Days post infection	Daily percentage risk of onward transmission* (%)	Infected person's state
1	0.00	Days 1-3: Infected,
2	0.00	not yet infectious
3	0.00	
4	16.78	Days 4-5: Infectious,
5	18.03	pre-symptomatic
6	17.07	Days 6-14: Infectious, symptomatic
7	14.52	
8	11.27	
9	8.10	
10	5.48	
11	3.55	
12	2.26	
13	1.46	
14	1.48	
Total	100	

*Percentages show when onward transmission might occur by the day of infectiousness
Sources: He *et al*. (*7*) and Ferretti *et al*. (*8*) See also COVIDTracer modeling tool manual (*3*).

Appendix Table 7. Epidemiologic parameters, values, and sources.

Parameter	Default Value	Source
Infected but not yet infectious period	3 d	CDC COVID-19 Pandemic Planning Scenarios (*13*)
Pre-symptomatic and contagious (infectious) period	2 d	He *et al*. (*7*), Ferretti *et al*. (*8*)
Symptomatic and contagious (infectious) period	9 d	He *et al*. (*7*), Ferretti *et al*. (*8*)
Basic Reproduction Number (R_0), original strain	2.5	CDC COVID-19 Pandemic Planning Scenarios (*13*)
% of cases that are asymptomatic	40%	CDC COVID-19 Pandemic Planning Scenarios (*13*)
Infectiousness of asymptomatic cases (relative to symptomatic cases)	75%	CDC COVID-19 Pandemic Planning Scenarios (*13*)

Appendix Table 8. Assumed* proportion of cases by age group and infection-to-hospitalization rate, default values in COVIDTracer and sources.

Age group (year)	% of Total Cases	Source	% of all cases admitted to hospital care	Source
0 to 17	15	CDC COVID Data	0.21	CDC COVID-19 Response Team (*5*),
18 to 64	55	Tracker (*15*)	2.17	Wu *et al*. (*6*)
65+	30		4.12	

*Derived September 2020 using sources available at that time.

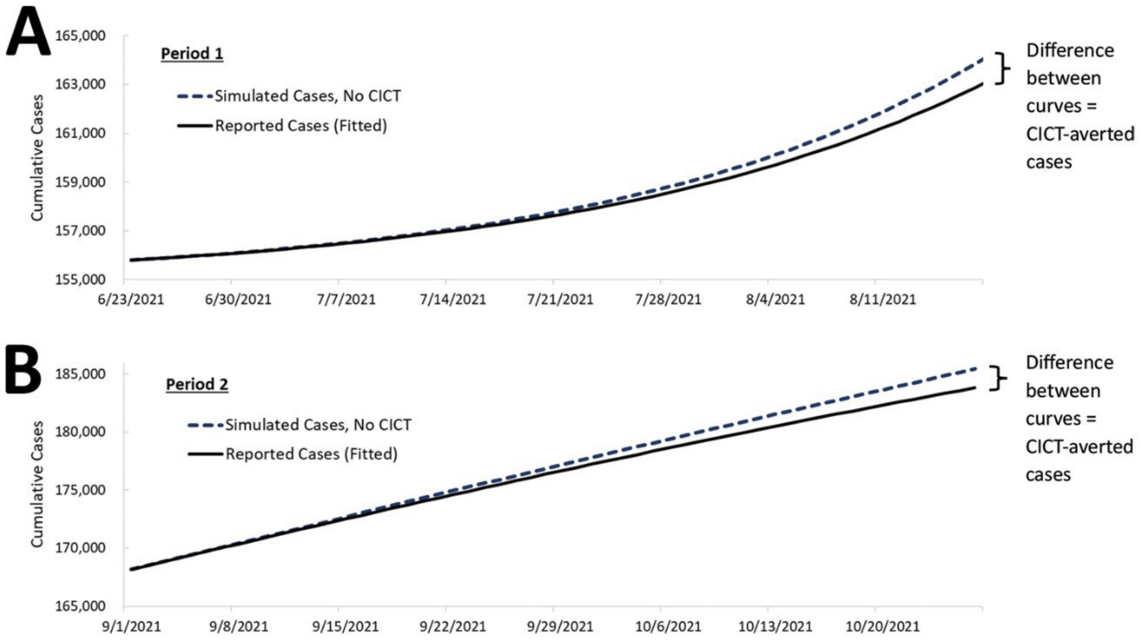

Appendix Figure 1. Epidemic curves fitted to reported COVID-19 case counts with case investigation and contact tracing (CICT), and estimated cases illustrating what might have occurred without CICT. The top panel (Period 1) illustrates the impact of CICT employing the original protocol and the bottom panel (Period 2) illustrates the impact of CICT after the protocol change occurred.

	Day 1	2	3	4	5	6	7	8	9	10	11	12	Day 13	Days from Exposure to Isolation
**Index Case**	Exposed			Contagious Period Begins		Symptom Onset		Tested			Case Interview	Begin Isolation		11
**Contacts** (Earliest possible exposure)				Exposed								Exposure Notification	Begin Quarantine	9
**Contacts** (Latest possible exposure)											Exposed	Exposure Notification	Begin Quarantine	2

Appendix Figure 2. Timing of COVID-19 case isolation and quarantine of contacts in Philadelphia, Pennsylvania, before the CICT protocol change, June 23 to August 17, 2021. We assumed a 5-day pre-symptomatic period. The Philadelphia Department of Public Health (PDPH) reported on average 2 days from symptom onset to specimen collection, 3 days from specimen collection to the case interview, and 4 days for contact notification before the CICT protocol change. The index case started showing symptoms on day 6 post-infection, underwent testing on day 8, and was interviewed by the health department on day 11. The contacts of the index case were exposed sometime between days 4 to 11 and were notified of their exposure on day 12. Therefore, the index case began isolation on day 12, and the contacts went

into quarantine on day 13 (based on the aforementioned assumptions). To calculate the days from contacts' exposure to their quarantine, we took the average of the maximum days a contact was infected (9 days, based on the earliest possible exposure) and the minimum days the contact could be infected (2 days, based on the latest possible exposure), and weighted each day span by the case's infectiousness on each of possible exposure day. The result was 5.9 days in this example. Subsequently, we calculated the average between 11 days (index case) and 5.9 days (contacts) as the number of days from exposure to isolation (for both cases and contacts), which totaled 8 days. This final value of 8 days represents one of the key CICT performance metrics, the number of days from exposure to isolation/quarantine.
